# Supplementary material for: Targeting Mechanisms of the DNA Damage Response (DDR) and DNA Repair by Natural Compounds to Improve cAT-Triggered Tumor Cell Death
Source: Molecules. 2022 Jun 1;27(11):3567. doi: 10.3390/molecules27113567 (PMC9182506; doi:10.3390/molecules27113567)
Supplement: Supplementary file 1 [file molecules-27-03567-s001.zip › molecules-1680101-supplementary.pdf]

Angenvoort et al - Supplementary Figures

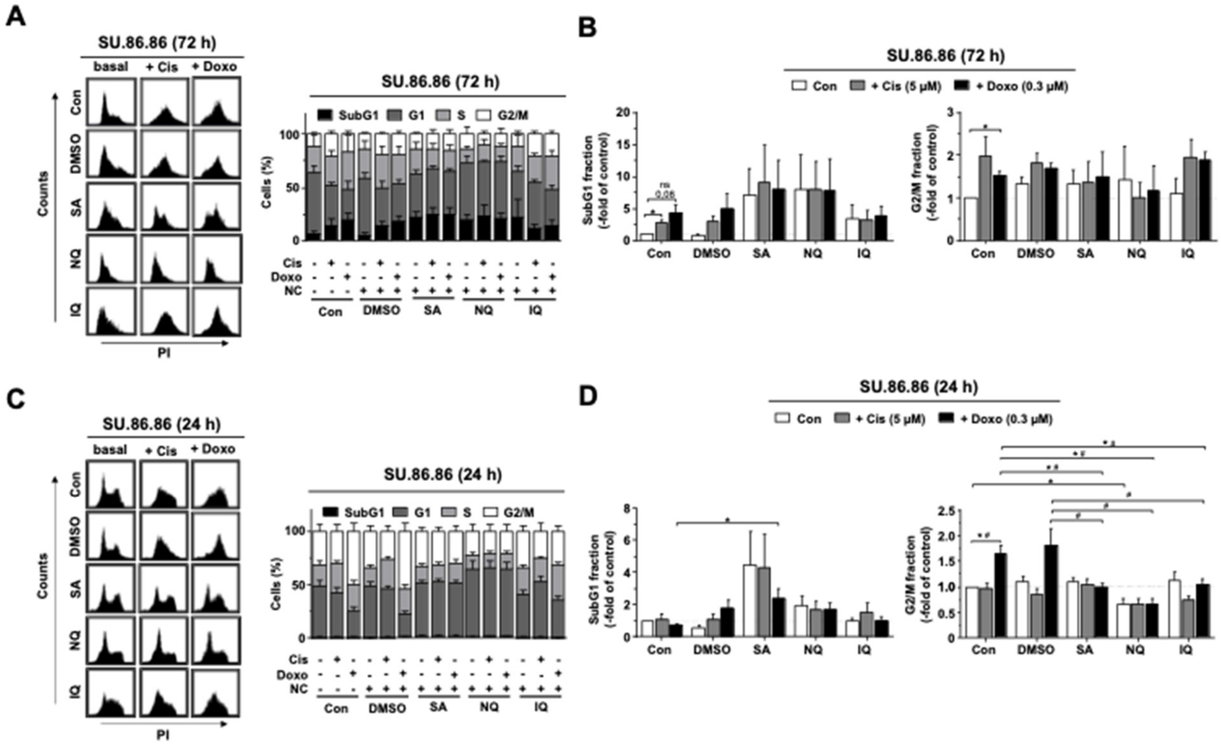

Supplementary Figure S1: Effect of NC on cell cycle distribution.

Logarithmically growing pancreatic carcinoma cells (SU.86.86) were treated with the various NC (SA, 3 μM; NQ, 5 μM; IQ, 1.5 μM). For the combination treatment, the cells were additionally exposed to cisplatin (5 μM) (Cis) or doxorubicin (0.3 μM) (Doxo). Cell cycle distribution was measured 24 h and 72 h later by flow cytometry.

A, C: Representative flow cytometry data and percentage of cells in the cell cycle as compared to non-treated control cells as analyzed 72 h (A) or 24 h (B) after drug addition. Shown are mean values + SEM obtained from three independent experiments.

B, D: Cells in SubG1 fraction or G2/M phase of the cell cycle as compared to non-treated control cells. Shown are mean values + SEM obtained from three independent experiments. *,#p≤0.05. Con, control; DMSO, dimethyl sulfoxide; IQ, 5-epi-ilimanquinone; NC, natural compound; NQ, 5-epi-nakijiquinone Q; PI, propidium iodide; SA, secalonic acid F.

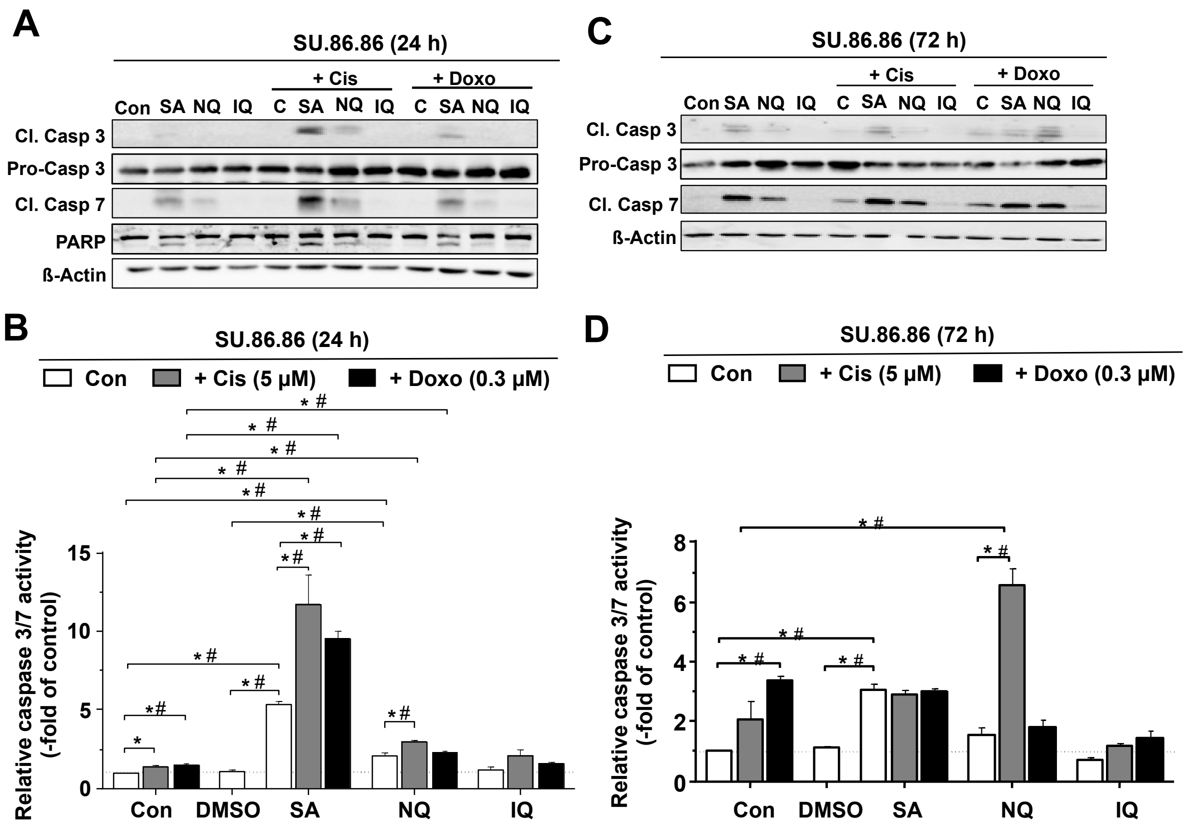

Supplementary Figure S2: Activation of caspase-3 and -7 by NC.

SU.86.86 pancreatic carcinoma cells were incubated with natural compounds only (SA, 3 µM; NQ, 5 µM; IQ, 1.5 µM) or in combination with cisplatin (Cis) or doxorubicin (Doxo) for 24 h (A, B) or 72 h (C, D) .

A, C: Protein level of cleaved pro-caspase-3 (Cl. Casp 3) and -7 (Cl. Casp 7) was analyzed by western blot analysis.

B, D: Caspase-3/7 activity was examined by use of the Apo-ONE® assay as described in methods. Quantitative data shown in B and D are the mean + SEM from three independent experiments each performed in triplicate. *,#p≤0.05. Con, control; IQ, 5-epi-ilimanquinone; NQ, 5-epi-nakijiquinone Q; SA, secalonic acid F.

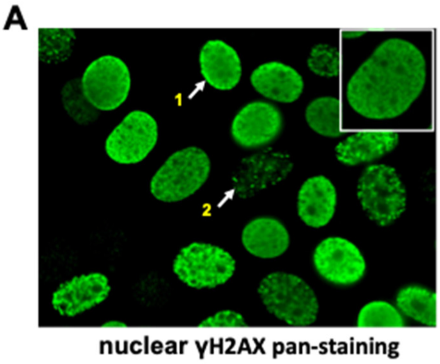

B

	0-25 foci/cell	26-50 foci/cell	≥ 51 foci/cell	pan-nuclear γH2AX
Con 4 h + 0 h	98 %	1 %	1 %	-
Cis 4 h + 0 h	61 %	32 %	7 %	-
Con 4 h + 24 h	99 %	1 %	-	-
Cis 4 h + 24 h	19 %	34 %	34 %	13 %
SA	71 %	26 %	3 %	-
Cis + SA	13 %	22 %	17 %	48 %
NQ	89 %	11 %	-	-
Cis + NQ	17 %	31 %	52 %	-
IQ	82 %	13 %	5 %	-
Cis + IQ	20 %	37 %	43 %	-

Supplementary Figure S3: Nuclear H2AX pan-staining after pulse treatment of BxPC3 cells with cisplatin followed by post-incubation with the natural compound SA.

A: Shown is a representative immunofluorescence picture observed after pulse treatment with cisplatin (Cis) (5 µM; 4 h) and further post-incubation with SA (3 µM, 24 h). Arrows point to cells with γH2AX pan-stained nucleus (arrow 1) or nucleus with distinct nuclear γH2AX foci (arrow 2).

B: The table depicts the percentage of low (0–25 γH2AX foci/cell), high (26–50 γH2AX foci/cell) and severely (≥ 51 γH2AX foci/cell) damaged cells as well as pan-stained cells. 50 nuclei were analyzed to calculate the percentages. Data shown are mean values from three independent experiments (n=3).

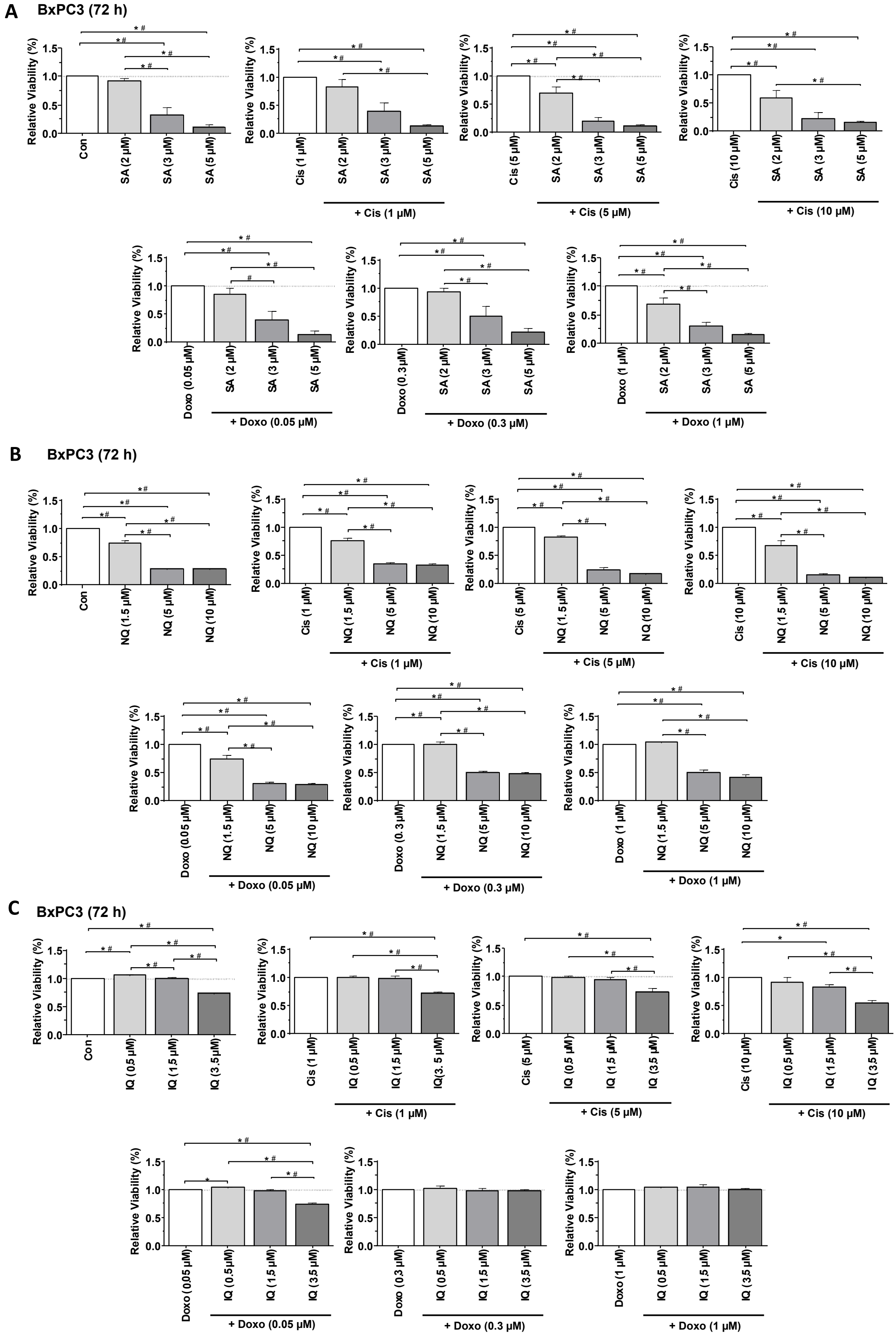
A
BxPC3 (72 h)
Relative Viability (%)
Con
SA (2 μM)
SA (3 μM)
SA (5 μM)
Cis (1 μM)
+ Cis (1 μM)
Cis (5 μM)
+ Cis (5 μM)
Cis (10 μM)
+ Cis (10 μM)
Doxo (0.05 μM)
+ Doxo (0.05 μM)
Doxo (0.3μM)
+ Doxo (0.3 μM)
Doxo (1 μM)
+ Doxo (1 μM)
B
BxPC3 (72 h)
NQ (1.5 μM)
NQ (5 μM)
NQ (10 μM)
C
BxPC3 (72 h)
IQ (0.5 μM)
IQ (1.5 μM)
IQ (3.5 μM)

D SU.86.86 (72 h)

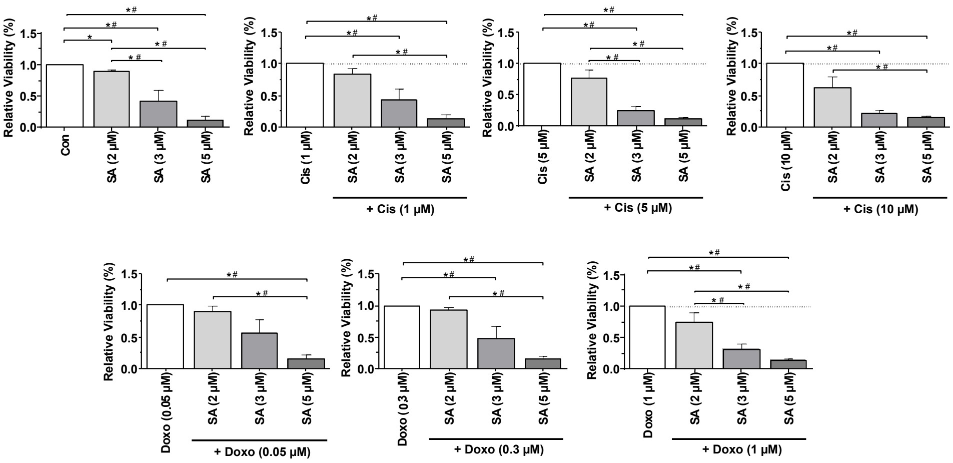

E SU.86.86 (72 h)

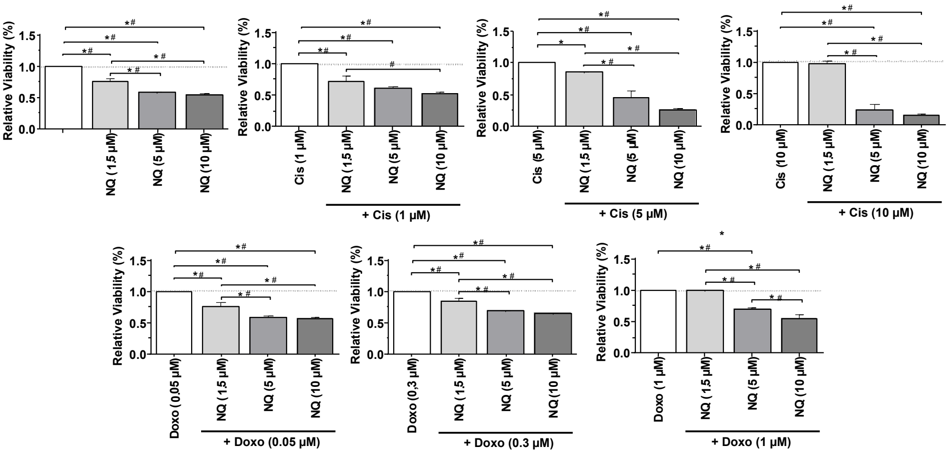

F SU.86.86 (72 h)

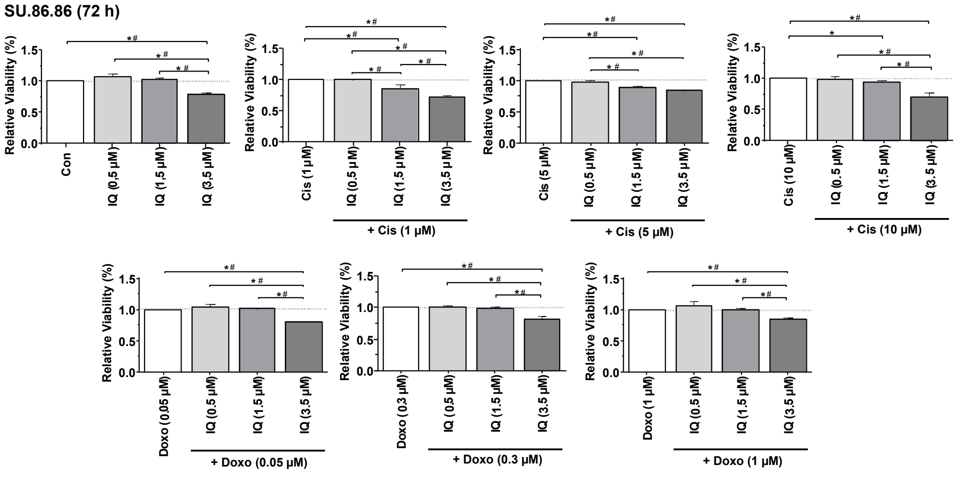

Supplementary Figure S4: Influence of combination treatment on cell viability.

In order to investigate the influence of the combination treatments on viability, BxPC3 **(A-C)** and SU.86.86 cells **(D-F)** were treated for 72 h with combinations of natural compounds and cisplatin (Cis) or doxorubicin (Doxo). In view of the respective IC_{20}, IC_{50} and IC_{80} of the various compounds reported in previous study [42], the following concentrations of the individual substances were used for calculating the combination index (CI): Cis (1, 5 and 10 μM); Doxo (0.05, 0.3 and 1 μM); SA (2, 3 and 5 μM); NQ (1.5, 5 and 10 μM); IQ (0.5, 1.5 and 3.5 μM). Viability was determined by the Alamar Blue® assay. The combination indices (CI) calculated from these extensive analyses are presented in Fig. 7 of

the manuscript. IQ, 5-epi-ilimanquinone; NQ, 5-epi-nakijiquinone Q; SA, secalonic acid F. *,p≤0.05 (Student´s t-test); #, p≤0.05 (One-way ANOVA).

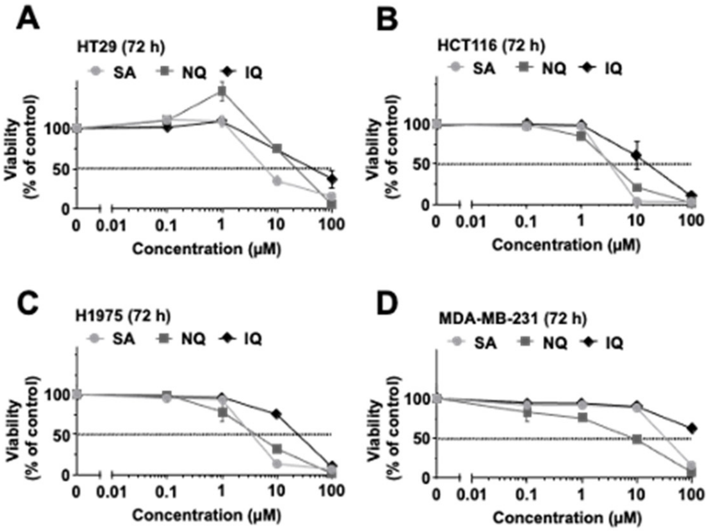

E

IC_{50} (72 h)		Natural compound (µM)		
		SA	NQ	IQ
Cell line	BxPC3	3	2	8
	SU.86.86	3	5	1.5
	HT29	5	20	40
	HCT116	3	3	20
	H1975	3	4	25
	MDA-MB-231	35	10	n.d.

Supplementary Figure S5: Effects of natural compounds on the viability of various types of human tumor cells.

A-D: To determine the IC_{50} of the natural compounds (NC), colon carcinoma cells (HT29 (A) and HCT116) (B) as well as lung carcinoma cells (H1975) (C) and triple negative breast cancer cells (MDA-MB-231) (D) were treated with concentrations between 0.1 and 100 µM for 72 h. NC were added 24 h after seeding. For all treatments, viability was measured using the Alamar Blue® Assay as described in methods. Cell viability data are presented as mean ± SEM from three independent experiments, each performed in quadruplicate.

E: Calculated IC_{50} for each compound and human tumor cell lines are listed in the table. IQ, 5-epi-ilimanquinone; NQ, 5-epi-nakijiquinone Q; SA, secalonic acid F. n.d., not determined.

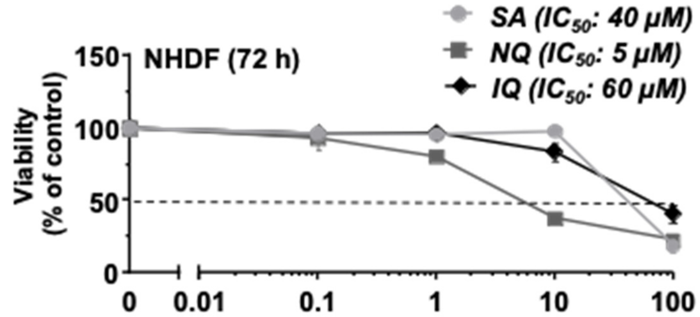

Supplementary Figure S6: Effect of natural compounds on the viability of non-malignant primary human fibroblasts.

To determine the IC_{50} of the natural compounds (NC) on non-malignant human cells, primary human fibroblasts (NHDF) were treated with different concentrations of the NC (0.1 - 100 µM) 24 h after seeding for 72 h. Afterwards, viability was measured using the Alamar Blue® Assay as described in methods. Cell viability data are presented as mean ± SEM from three independent experiments (n=3), each performed in quadruplicate. SA, secalonic acid F; NQ, 5-epi-nakijiquinone Q; IQ, 5-epi-ilimanquinone.

Supplementary Table S1: Primer sequences used for qRT-PCR mRNA expression analyses of selected genes involved in DDR and DNA-repair, apoptosis, oxidative stress, general stress response and transport.

Target Gene	Sequence forward primer	Sequence reverse primer
ACTB	TGGCATCCACGAAACTACC	GTGTTGGCGTACAGGTCTT
ATP7A	AGGCAGAAGTAAGGTATAATCC	CTCACAACAAGTTCCAAAAC
BAX	ATCAGATGTGGTCTATAATG	CAGAAGGCACTAATCAAG
BCL2	CGACTCCTGATTCATTGG	TCTACTTCCTCTGTGATGT
BIRC5	ACCTGAAAGCTTCCTCGACA	TAACCTGCCATTGGAACCTC
BRCA1	AAGACTTCTACAGAGTGAA	CAGTTCCAAGGTTAGAGA
BRCA2	AACAACAATTACGAACCAA	AACATTCCTTCCTAAGTCTA
Calpain	AGACCATGTTCCGATTTTTC	TGCAACCACTTAAACAAGTC
Casp6	AAGGATATTATTCTCACCGGG	TGAGAAACTTTCCTGTTCAC
Casp9	CTCTACTTTCCCAGGTTTTG	TTTCACCGAAACAGCATTAG
CHK1	CCACCTCTTCATAACAACAA	TAAATCACAATCGCCACTC
CHK2	GCACTGTCACTAAGCAGAAAT	AGGCACCACTTCCAAGAG
CTR1	TGATGCCTATGACCTTCTAC	GAATGCTGACTTGTGACTTAC
DYRK1VB	TCAGCATCATTCACTGCG	ATATACTGGTAGATCCTCTGG
ERBB2	CCAGCCTGAATATGTGAAC	CCCCAAAGGCAAAAACG
ERCC1	AGGAAGAAATTTGTGATAC	TGTGTAGATCGGAATAAG
FASL	CAATCTTACCAGTGCTGAT	AATCCCAAAGTGCTTCTC
FASR	TTATCTGATGTTGACTTGAGTAA	GGCTTCATTGACACCATT
GADD45A	ATCCACATTCATCTCAAT	GTAACTACAAAGGTATTTCA
GAPDH	CATGAGAAGTATGACAACAG	ATGAGTCCTTCCACGATA
GPX1	GCCAAGAACGAAGAGATT	TCGAAGAGCATGAAGTTG
GSTM1	ACTATCCTTCGTGAACATC	AGACACAACCACTAACAG
HMOX1	CAACAAAGTGCAAGATTC	AGAAAGCTGAGTGTAAGG
HSP90AA1	AGTTATCCTACACCTGAA	CATCATCGCTTACTTCTT
HSPA1B	GGACTTTGACAACAGGCT	GCTTGTTCTGGCTGATGT
MLH1	AAGTTGTTGGCAGGTATT	GGTAGTGTCCTAACATCAG
MRE11	GCCTGTCCAGTTTGAAAT	GGTTGCCATCTTGATAGTT
MRP2	AAATTGCTGATCTCCTTTGC	GATAGCTGTCCGTACTTTTAC
MSH2	CTTCTTCTGGTTCGTCAGTATAGA	ATCATTCTCCTTGGATGCCTTAT
MT1A	TGGCTCCTGCACCTGCAC	GAGCAGCAGCTCTTCTTGC
P53	AGCACTAAGCGAGCACTG	ACGGATCTGAAGGGTGAAA
POLH	GGATAGAATACATGGGTGAAC	TCCTGGGAAGTTCTTACTAC
RAD51	AATTAGTTCCAATGGGTTT	TGAAGTAGTTTGTCAAGC
TP63	ATTGTTCTCCGTTCGTTGATA	GTAAGGGTCAGGGCAGTA
VDAC	CACACTAATGTGAATGACGG	AGGGTCAATCTGATACTTGG
XAF1	GATCCACTTTTGATGTCAGAG	CCTGATGTTGATTTAGGATCG
XRCC3	CATTGTTCTGTCTTTCCT	CTCCTTTACCGATTTCAG

Supplementary Table S2: NC-modulated DDR of pancreatic carcinoma cells.

Activation of mechanisms of the DDR following mono- or combined-treatment of pancreatic carcinoma cell lines (BxPC3 and SU.86.86) with the natural compounds (NC) and cisplatin (Cis) or doxorubicin (Doxo) were analyzed by Western blot analysis using phospho-specific antibodies of selected DDR-related factors, followed by densitometrical analyses (original data see Fig. 1). The impact of the NC on the phosphorylation (=activation) status of the selected DDR factors was related to that of the corresponding Cis or Doxo mono-treated controls (+Cis, +Doxo), which were set to 1.0.
0: no effect (>0.7≤1.5-fold); +: stimulatory effect (>1.5≤3-fold); ++: strong stimulatory effect (>3-fold); -, weak inhibitory effect (>0.3<0.7-fold); -- strong inhibitory effect (≤0.3-fold).

		BxPC3 (24 h)		SU.86.86 (24 h)	
**NC**	**DDR factor**	**+ Cis[1]**	**+ Doxo[1]**	**+ Cis[1]**	**+ Doxo[1]**
**SA**	**pATM**	0	0	0	0
	**pATR**	0	0	0	0
	**pRPA32**	0	0	++	++
	**pCHK1**	- -	- -	- -	- -
	**pCHK2**	-	-	- -	- -
	**pKAP1**	- -	-	++	-
	**pp53**	- -	- -	- -	- -
	**γH2AX**	++	++	++	++
**NQ**	**pATM**	0	-	0	-
	**pATR**	0	0	0	0
	**pRPA32**	++	+	++	++
	**pCHK1**	- -	- -	- -	0
	**pCHK2**	-	0	-	- -
	**pKAP1**	+	0	++	+
	**pp53**	0	0	0	0
	**γH2AX**	++	++	++	++
**IQ**	**pATM**	0	-	0	0
	**pATR**	0	+	0	-
	**pRPA32**	-	0	+	0
	**pCHK1**	0	++	0	0
	**pCHK2**	0	0	0	- -
	**pKAP1**	-	0	0	0
	**pp53**	0	0	0	0
	**γH2AX**	0	+	0	+

Supplementary Table S3: Summary of NC-induced alteration in mRNA expression levels of susceptibility-related genes.

Overview of the results obtained via qRT-PCR analyses (original data see Fig. 6A). The analyzed genes are classified into genes involved in DDR and DNA-repair, apoptosis, oxidative stress, general stress response and transport. -, --: weak, strong downregulation; +, ++: weak, strong upregulation; 0: no effect as compared to the untreated control group; nd.: not detectable.

	BxPC3 (24 h)			
	**Gene**	**SA**	**NQ**	**IQ**
DDR and DNA repair	*BRCA1*	--	0	0
	BRCA2	--	0	0
	CHK1	-	0	0
	CHK2	-	0	0
	ERCC1	0	0	0
	GADD45A	++	++	+
	MLH1	-	0	+
	MRE11	--	0	0
	MSH2	-	0	0
	P53	-	0	0
	POLH	0	0	0
	RAD51	--	0	0
	TP63	0	0	+
	XRCC3	-	0	0
Apoptosis	*BAX*	0	0	0
	BCL-2	0	++	0
	BIRC5	0	0	0
	Calpain	-	+	0
	Caspase 6	-	0	0
	Caspase 9	-	++	0
	DYRK1VB	-	++	0
	FASL	++	nd.	nd.
	FASR	--	+	0
	VDAC	0	0	0
	XAF1	-	0	+
Oxidative Stress	*GPX1*	0	+	0
	GSTM1	0	0	-
	HMOX1	0	++	+
	MT1A	0	++	+
Stress response	*ERBB2*	--	+	0
	HSP90	0	0	0
	HSPA1B	++	0	0
Transport	*ATP7A*	0	++	++
	CTR1	0	+	0
	MRP2	0	0	0
